# Single-Photon Counting with Semiconductor Resonant Tunneling Devices

**DOI:** 10.3390/nano12142358

**Published:** 2022-07-09

**Authors:** Andreas Pfenning, Sebastian Krüger, Fauzia Jabeen, Lukas Worschech, Fabian Hartmann, Sven Höfling

**Affiliations:** 1 Stewart Blusson Quantum Matter Institute, University of British Columbia, Vancouver, BC V6T 1Z4, Canada; 2 Technische Physik, Wilhelm-Conrad-Röntgen-Research Center for Complex Material Systems, Würzburg-Dresden Cluster of Excellence ct.qmat, University of Würzburg, 97074 Würzburg, Germany; sebastian.krueger@physik.uni-wuerzburg.de (S.K.); fauzia.jabeen@physik.uni-wuerzburg.de (F.J.); lukas.worschech@uni-wuerzburg.de (L.W.); fabian.hartmann@physik.uni-wuerzburg.de (F.H.); sven.hoefling@physik.uni-wuerzburg.de (S.H.)

**Keywords:** single-photon detectors, resonant tunneling diode, photon counting, III–V semiconductor devices

## Abstract

Optical quantum information science and technologies require the capability to generate, control, and detect single or multiple quanta of light. The need to detect individual photons has motivated the development of a variety of novel and refined single-photon detectors (SPDs) with enhanced detector performance. Superconducting nanowire single-photon detectors (SNSPDs) and single-photon avalanche diodes (SPADs) are the top-performer in this field, but alternative promising and innovative devices are emerging. In this review article, we discuss the current state-of-the-art of one such alternative device capable of single-photon counting: the resonant tunneling diode (RTD) single-photon detector. Due to their peculiar photodetection mechanism and current-voltage characteristic with a region of negative differential conductance, RTD single-photon detectors provide, theoretically, several advantages over conventional SPDs, such as an inherently deadtime-free photon-number resolution at elevated temperatures, while offering low dark counts, a low timing jitter, and multiple photon detection modes. This review article brings together our previous studies and current experimental results. We focus on the current limitations of RTD-SPDs and provide detailed design and parameter variations to be potentially employed in next-generation RTD-SPD to improve the figure of merits of these alternative single-photon counting devices. The single-photon detection capability of RTDs without quantum dots is shown.

## 1. Introduction

High-speed and high-efficiency single-photon detectors (SPDs) are a fundamental requirement for a broad range of applications, particularly in optical quantum information technologies, such as quantum key distribution, boson sampling, quantum sensing or optical quantum computing [1,2,3]. Evidentially, each of these applications has their own particular needs in terms of detector performance. For example, quantum key distribution does not necessarily rely on photon-number resolution, but rather on low dark count rates as well as low deadtime and timing jitter. Linear optical quantum computing approaches require overall system efficiencies exceeding 2/3, and thus detector efficiencies of over 90% for its successful implementation [3,4], whereas quantum computing approaches using the in-phase and quadrature of the electromagnetic field amplitude also rely on photon-number resolution (PNR) [5,6,7].

Overall, a growing demand for ever better detector performance has resulted in a variety of novel optoelectronic devices and device refined device concepts. By far the most impactful single-photon detector technology of the past decade are superconducting nanowire single-photon detectors (SNSPDs) [8,9]. They offer the highest detection efficiency, low dark counts, and excellent timing resolution, which justifies their requirement of cryogenic operation ideally in the sub-Kelvin regime [9]. As with other click-detectors, SNSPDs can be limited by their deadtime and generally lack the capability of photon-number resolution.

Another emerging class of single-photon detectors are semiconductor resonant tunneling diodes (RTDs) [10]. RTDs are nanoelectronic devices that are most renowned for their peculiar current–voltage characteristics, which exhibit a region of negative differential conductance (NDC) [11,12,13]. The applications of RTDs comprise a broad range from electronic THz emitters and receivers to artificial neurons [14,15,16,17]. When applied as sensors, it is exploited that the resonant tunneling current is sensitive to even the smallest changes in the local electrostatic environment. As such, RTD photodetectors can be operated as low-noise and high-speed amplifiers of small optically generated electrical signals [18,19]. The arguably most impressive feature of RTD photodetectors is their capability to resolve the presence of individual photogenerated charge carriers, which was first reported by *Blakesley* et al., who showed that the resonant tunneling current across a double barrier quantum well can be modulated by the entrapment of individual photogenerated holes within an adjacent quantum dot (QD) layer [10].

RTD single-photon detectors boast, at least in theory, a variety of desirable characteristics when compared to conventional SPDs. In contrast to SNSPDs and SPADs, a photon detection event does not cause a breakdown (quenching) of the current flow, which makes RTD-SPDs inherently photon-number resolving and deadtime free in the classical sense. Remarkably, the PNR can even be achieved at elevated temperatures [20].

Combining our previous work [21,22], and current experimental results, the present overview on RTD-SPDs with a focus on the state of the art, the underlying device physics, limitations, and recent developments is structured and intended as follows:

In Section 2. *The Fundamentals of Resonant Tunneling Single Photon Detection,* we discuss the underlying physics and working principles of RTD SPDs.

In Section 2.1. *Resonant Tunneling Diodes,* we briefly introduce the RTD as nanoelectronic device on the basis of the example of a III-V semiconductor double-barrier quantum well.

In Section 2.2*. The Photodetection Mechanism and Operational Modes*, we revisit the RTD photodetection mechanism and elaborate on how the NDC region enables various operational modes.

In Section 2.3. *Single-Photon Detection with Resonant Tunneling Diodes*, we provide a theoretical framework on the pre-requisites required to achieve single-photon detection that can be used for device optimization and performance predictions. The model is then experimentally checked and verified.

In Section 3. *Practical Device Design Considerations,* we elaborate on various design considerations in order to optimize future detector performance.

In Section 3.1. *Quantum Dots are not a Necessity*, we demonstrate that photon-counting with RTDs is possible even without QDs as minority charge carrier traps.

In Section 3.2. *Detector Architectures and Device Design*, we revisit various RTD photodetector architectures, and compare their respective advantages and disadvantages.

In Section 3.3. *Strategies against the Quantum Efficiency Dilemma–Cavity-Enhanced Detectors*, we provide a pathway to near-unity detection efficiencies in RTD-SPDs based on a carefully designed heterostructure integrated into an optical distributed Bragg reflector (DBR) cavity.

In the final Section 4. *Quantifying the Device Performance of RTD-SPD*, we assess and compare the performance of RTD single-photon detectors in terms of spectral range, deadtime, dark count rate, detection efficiency, timing jitter, and the ability to resolve photon number.

## 2. The Fundamentals of Resonant Tunneling Single-Photon Detection

### 2.1. Resonant Tunneling Diodes

RTDs are structurally rather simple two-terminal nanoelectronic devices that make use of two quantum-mechanical effects: energy quantization due to electronic confinement and tunneling through a potential barrier. Notable, these quantum mechanical effects in RTDs can be observed even at room temperature and above.

**Figure 1:**
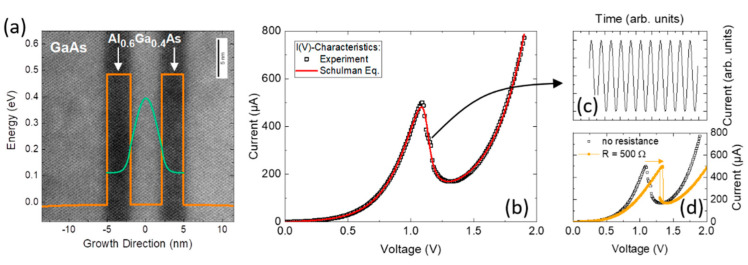
Resonant tunneling diode fundamentals. (**a**) Transmission electron microscopy image (TEM) of an AlGaAs/GaAs double barrier quantum well resonant tunneling diode. The conduction band profile is shown as orange line. The two Al_0.6_Ga_0.4_As barriers sandwich the GaAs quantum well, which lead to energy quantization in the conduction band. (**b**) Typical RTD current–voltage characteristic. Experimental data is shown as black squares. The characteristics calculated from *Schulman’s* model is displayed as red line. (**c**) When operated in the negative-differential conductance (NDC) region, the RTD can provide electrical gain and drive an oscillator circuit. (**d**) For series resistance values larger than the inverse absolute value of the NDC, the RTD exhibits a bistable input-output characteristics and can thus be operated as bistable switch. Figure 1 was originally published in Ref. [22].

Figure 1a shows a transmission electron microscopy (TEM) image of an exemplary Al_0.6_Ga_0.4_As/GaAs DBQW. Dark contrast refers to the Al-rich barriers. The two 3 nm tick Al_0.6_Ga_0.4_As barriers are sandwiching a 4 nm thick GaAs quantum well. The conduction band profile is shown as orange line and overlayed with the TEM image. Due to the confinement potential of the two Al_0.6_Ga_0.4_As barriers, quantization of the energy occurs. The DBQW ground state wavefunction absolute square ${\left|\Psi \right|}^{2}$ is depicted as green line. Similar to an optical resonator, for incident electrons with an energy resonant to the DBQW ground state energy, the DBQW becomes transparent, and the electrons can tunnel from the emitter to the collector side. Please note that even though here we show an example of an Al(Ga)As/GaAs resonant tunneling structure, RTDs and RTD photodetectors can be created in a variety of materials, e.g., InGaAs, InP, Si/Ge, III-nitride, and the 6.1-Å semiconductor family.

The energy of electrons in the emitter side with respect to the ground and/or excited energy states can be tuned by applying a bias voltage between emitter and collector contact. Figure 1b shows a typical current–voltage (I(V)) characteristic of an AlGaAs/GaAs DBQW RTD. Experimental data are shown as black squares. In terms of empirical data analysis and modeling, the Schulman’s physics-based current–voltage equation can be easily modified to include various physical effects and is a simple and intuitive model to simulate the current–voltage characteristic of RTDs [23]. The I(V) characteristic modeled after Schulman’s equation is shown as red solid line. As the bias voltage increases, the current increases exponentially until a local maximum is reached at a resonant bias voltage of V=1.1 V. At this particular voltage, there is a maximum energetic overlap between the electron emitter population and the DBQW ground state. Further increasing of the bias voltage detunes the respective emitter and ground state energies, resulting in the feature of negative differential conductance, that is, a reduction in current despite an increase in the bias voltage.

In terms of RTD-SPDs, the NDC region gives rise to a higher functionality as multiple operation modi can be used dependent on the external circuit or biasing condition. For example, the electrical gain provided by the NDC region can be used to drive high-speed oscillator circuits (see Figure 1c). With fundamental oscillation frequencies up to f=2 THz [24], RTDs are among the fastest semiconductor devices. Interestingly, in conjunction only with a parasitic and passive resistance in series, the RTD can be operated as a bistable logic device (see Figure 1d). Later in this review, we will discuss in more detail how the NDC region can be exploited in terms of photodetection. For a recent review of RTD THz oscillators and detectors, see Refs. [15,25], and for a comprehensive introduction into negative differential conductance devices and circuits, please refer to Ref. [26].

### 2.2. The Photodetection Mechanism and Operational Modes

RTD photodetectors are based on the principle that the resonant tunneling current can be influenced by introducing changes to their local electrostatic potential. Such a change in the local electrostatic potential can be induced by capturing optically generated minority charge carriers in the vicinity of the resonant tunneling structure at the collector-sided depletion region [27]. These entrapped minority charge carriers lead to an additional voltage drop across the resonant tunneling structure, which results in a shift of the current–voltage characteristics towards smaller bias voltages [27,28]. The fundamentals of the RTD photosensitivity have been studied in detail [18,19,29], and been applied to different material systems to cover the visible [30], near-infrared [31,32], and even mid-infrared spectral region [33,34,35].

As a novel class of single-photon detectors, the detailed impact of the RTD-SPD heterostructure layer material properties on the device performance remains largely unexplored. The choice of a single design parameter of the epilayer stack typically influences multiple device parameters simultaneously. The doping profiles of the emitter and collector region can be considered a prime example. The high doping densities of the emitter side up to the resonant tunneling structure with a spacer layer of only a few nm will lead to increased current densities as well as lower operation voltages. However, the degradation of the peak-to-valley current ratio might be expected. In particular, the Geiger mode operation profits from large PVCRs. Conversely, the collector side depletion region determines the detection efficiency of the RTD-SPD. To ensure a maximum percentage of photogenerated minority charge carriers is captured for the accumulation in vicinity of the resonant tunneling structure, doping should be kept low and the collector side depletion region should comprise a lower bandgap energy absorption layer.

**Figure 2:**
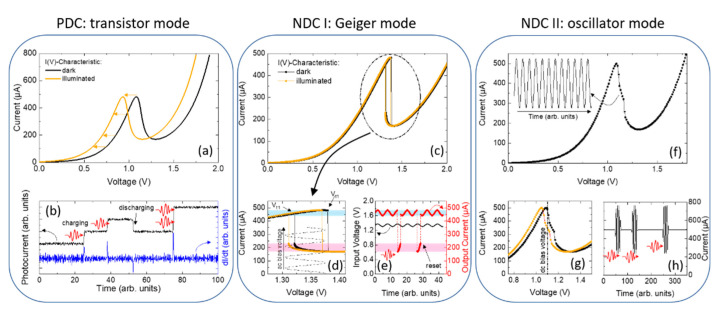
Operational modes of RTD-PDs. (**a**,**b**) PDC: transistor mode. (**c**–**e**) NDC I: Geiger mode. (**f**–**h**) NDC II: oscillator mode. All operational modes rely on the accumulation of photogenerated minority charge carriers in the vicinity of the DBQW. Due to the additional voltage drop, the current–voltage characteristics are shifted to lower voltages. In PDC: transistor mode, the RTD-SPD is biased at a fixed voltage and the resulting photocurrent is caused by the shift of the current–voltage characteristic due to the charging or discharging of holes in the vicinity of the DBQW. Incoming photons thus result in a step-like increase in the photocurrent, while the discharging process (i.e., the hole tunnels through the DBQW) leads to a decrease in the photocurrent. NDC I: Geiger mode and NDC II: oscillator mode, on the other hand, work on current variations that are triggered by light in combination with the designed oscillator circuit and/or an external ac driving frequency. The RTD itself operates as an additional amplifier of small signals. Figure 2 was originally published in Ref. [22].

There are numerous ways an RTD photodetector can be operated. The mode of operation determines how the RTD photodetector is controlled and read out. Figure 2 shows a comparison of three operational modes: (i) the phototransistor mode, (ii) the Geiger mode, and (iii) as an optically controlled oscillator.

#### 2.2.1. Phototransistor Mode

The most common way to operate an RTD photodetector is in the phototransistor mode, which is presented in Figure 2a,b. Figure 2a shows the RTD I(V) characteristic in the dark (black line) and under illumination (orange line). Due to accumulated photogenerated minority charge carriers adjacent to the DBQW, the I(V) characteristic shifts towards smaller voltages. For the phototransistor mode, the RTD is typically operated within one of the positive differential conductance (PDC) regions, even though this is not a strict requirement. A constant voltage is applied, and the current is measured. The voltage shift under illumination causes a measurable increase in the current flow. The current increment induced by an individual photogenerated minority charge carrier must be greater than the current noise to enable photon counting. RTDs with sufficiently small tunneling junction areas can meet this requirement [21]. A simulation of the photocurrent-time trace is shown in Figure 2b as a black line. The capture events of the photogenerated charge carriers adjacent to the DBQW cause a discrete step-like increase in the current. Discharging events result in a step-like decrease in the current. If two photogenerated charge carriers are simultaneously captured, they are apparent in a step-like current increase of about twice the height. This implies that RTD single-photon detectors are inherently photon-number resolving. Furthermore, RTDs operated in the phototransistor mode do not suffer from a deadtime following a detection event, in which the detector is reset to its initial. With high-bandwidth-differentiating electronics, the current steps can be converted to short spikes (see Figure 2b, blue line) that are compatible with conventional counting electronics [10].

#### 2.2.2. Geiger Mode

One of the detection modes that exploits the RTD’s unique NDC region is the Geiger mode (presented in Figure 2c–e). Choosing a circuit with an adequate series resistance introduces an extrinsic bistability to the RTD current–voltage characteristics as shown in Figure 2c. The extrinsic bistability exhibits a distinct high-current state and low-current state with the threshold voltages $V_{t1}$ and $V_{t2}$ for the up-sweep and down-sweep, respectively. In the present example, the series resistance is $R_{s}=600$ Ω, $V_{t1}=1.38$ V, and $V_{t2}=1.32$ V, which results in a hysteresis width $\Delta V_{Hys}=0.06$ V. A series resistance might not be required if the RTD exhibits an intrinsic bistability. However, the control of the width of the extrinsic bistability via an external resistor is better controllable. A zoom-in of the hysteresis is given in Figure 2d. The high-current state and the low-current state are highlighted by the blue- and red-shaded regions, respectively. Under illumination, the threshold voltages shift towards lower voltages, which enables to operate the RTD as a bistable optoelectronic switch. In contrast to the phototransistor mode, an additional ac component of the bias voltage, $V\left(t\right)=V_{dc}+V_{ac}\left(t\right)$, is required to reset the RTD after a photon-induced switching event. The ac component is a periodic function, e.g., $V_{ac}\left(t\right)=V_{0}\cdot \sin\left(\omega t\right)$ with an amplitude greater than the hysteresis width, i.e., $V_{0}≳1/2\cdot \Delta V_{Hys}$. The dc component $V_{dc}$ is chosen to be off-center so that the maximum of V(t) is below $V_{t1}$; hence, $V_{dc}≲\left(V_{t1}+V_{t2}\right)/2$. In the dark, the RTD remains in the high-current state. Under illumination, photogenerated minority charge carriers captured for accumulation lead to a shift of $V_{t1}$ and $V_{t2}$ towards smaller voltages by ΔV. If the photoinduced voltage shift exceeds $\Delta V>V_{t1}-\left(V_{dc}+V_{0}\right)$, the maximum of V(t) is found above $V_{t1}$, which triggers a sharp transition from the high-current to the low-current state (see Figure 2e). The transition from high- to low-current state can be measured without the need for additional amplifiers. The reset is ensured by the condition $V_{0}≳1/2\cdot \Delta V_{Hys}$. The switching time is given by the sum of the RC time and the quasibound-state lifetime, which enables switching at the picosecond scale [36]. The room temperature operation of the Geiger mode in the few-photon regime has been achieved [37], but the distinct single-photon detection still needs to be demonstrated.

The Geiger mode does not allow for the determination of the arrival time of a single photon. Instead, it counts the number of incident photons within a certain time bin given by the periodicity of the ac voltage. Photon-number resolution has not yet been demonstrated, but can be encoded in the transition time. Dependent on the number of accumulated minority charge carriers, the bistable transition occurs at different timescales within the ac modulation and the counting appears in the timing of the pulse rather than the amplitude. It is important that, during the operation cycle, the RTD is returned into its initial state. Otherwise, left-over minority charge carriers will lead to false detection events.

#### 2.2.3. Oscillator Mode

Similar to the Geiger mode, the oscillator mode is enabled by the RTD’s negative-differential conductance (see Figure 2f–h). To operate the RTD in the oscillator mode, it is biased in the NDC region with a constant voltage just below the threshold to the oscillatory state. Under illumination and when the current-voltage characteristics shifts to lower bias voltages, the RTD is excited into the oscillatory regime and short radio-frequency bursts are excited [38,39]. Thus, the RTD needs to be biased just before the NDC region so that the optical excitation will shift the RTD into the NDC region. The oscillator regime has recently been demonstrated with an RTD photodetector that oscillates at a carrier frequency of f=79 GHz [39]. This scheme could be used as interface between optical communication networks and THz terminals, or even as a method of contactless read-out. Instead of short radio-frequency bursts, it is possible to excite non-oscillating spiking [40]. Please note, that in the oscillator mode the capability to detect single photons has yet to be demonstrated experimentally.

### 2.3. Single-Photon Detection with Resonant Tunneling Diodes

In the following, we focus on the phototransistor mode to derive the best device design parameters and compare the theoretical framework with the experimental results.

The capability of single-photon detection in RTDs is tied to the capability to detect individual photogenerated minority charge carriers. That is, the photocurrent induced by an individual captured minority charge carrier must be larger than the current noise, $\Delta I_{1}>i_{noise}$. Considering shot noise, this condition can be rewritten as:

(1) $$\Delta V_{1}\cdot \frac{\partial j_{RTD}\left(V\right)}{\partial V}\cdot A_{RTD}>\sqrt{2q_{0}\cdot j_{RTD}\left(V\right)\;A_{RTD}\cdot F\left(V\right)\cdot \Delta f}$$

where $\Delta V_{1}$ is the photoinduced voltage shift caused by a single trapped minority charge carrier, $j_{RTD}\left(V\right)$ is the current density, $A_{RTD}$ is the tunnelling junction area, and Δf is the bandwidth. For RTDs, the shot noise itself is a complex issue as it is not purely Poissonian. The rich device physics of RTDs exhibit a highly nonlinear noise characteristic that, dependent on the bias voltage, can vary from sub- to super-Poissonian. The shot-noise values can either be enhanced or reduced from its purely Poissonian value, which is accounted for by the Fano-factor F. Due to the Pauli exclusion principle and Coulomb repulsion, F(V) can be as low as 0.5 (in resonance) or increase up to F≫6 (in the NDC region) [41,42].

In a first order approximation and assuming that capturing a photogenerated minority charge carrier is similar to charging of a plate capacitor, $\Delta V_{1}$ can be considered a constant independent of V that is determined by the *RTD* material and heterostructure design: [18,28]

(2) $$\Delta V_{1}\approx \frac{2\epsilon_{W}l_{B}+\epsilon_{B}l_{W}}{\epsilon_{W}\epsilon_{B}}\cdot \frac{q_{0}}{n_{RTD}}\cdot \frac{1}{A_{RTD}}=\gamma_{RTD}\cdot \frac{1}{A_{RTD}}$$

where $\epsilon_{W}$ and $\epsilon_{B}$ are the electric permittivities of the well and barrier materials, respectively. The barrier width is denoted by $l_{B}$ and the quantum well width by $l_{W}$, and $q_{0}$ is the elementary charge and $n_{RTD}$ is the *RTD* leverage factor. The *RTD* leverage factor determines the efficiency with which the applied bias voltage shifts the energy level(s) of the quantized state(s) within the double-barrier quantum well with respect to emitter states. This takes into account the voltage drops across the various layers, interfaces and contacts. The $\gamma_{RTD}$ parameter is introduced for simplification. Notably, we find that $\Delta I_{1}$ is independent from $A_{RTD}$. Solving for $A_{RTD}$ allows to determine the maximal device area that will allow for single-photon detection:

(3) $$A_{RTD}<\frac{\gamma_{RTD}^{2}}{2q_{0}\cdot j_{RTD}\cdot \Delta f}\cdot {\left(\frac{\partial j_{RTD}}{\partial V}\right)}^{2}$$

Thus, via Equations (1)–(3), we can evaluate the maximal device areas, RC limitations, operational speed, step height of RTD-SPDs, and the expected signal-to-noise ratio. Please note that we restrict our derivation to shot-noise only, which is dominant at in high-speed frequencies far of the 1/f noise contribution.

**Figure 3:**
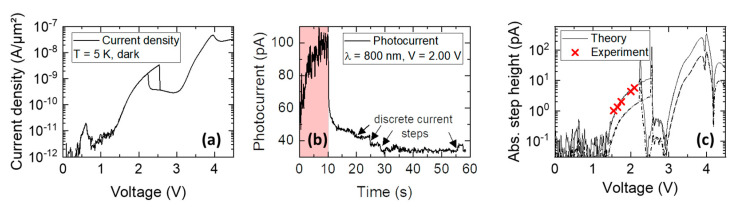
(**a**) Current density-voltage characteristic of an RTD-SPD with a 10 nm thick quantum well. Three resonances can be observed. (**b**) Background-corrected photocurrent taken at V=2.00 V. The red highlighted region marks the time when the laser was on (light power of 100 fW). During this time, the large fluctuations in the photocurrent indicate multiple photon detection events that are merged because of the time resolution of the setup. (**c**) Theoretical and experimental determination of the step height of a single charging event versus voltage. As the voltage is increasing, the current step height increases as well due to the increase in the differential conductance.

Figure 3 shows the operation of an RTD-SPD at a temperature of T=5 K. The RTD-SPD consists of two 10 nm thick Al_0.3_Ga_0.7_As barriers, a 10 nm thick GaAs quantum well, and self-assembled InGaAs quantum dots grown 4 nm after the DBQW. The sample was grown on an n-type Si-doped GaAs substrate with $n=3\times 10^{18}$ cm^−3^. The emitter (bottom) contact layer is formed by 340 nm of n-type-doped GaAs with a decreasing doping concentration of $n=3\times 10^{18}$ cm^−3^ to $1\times 10^{17}$ cm^−3^. The DBQW is separated from the emitter contact region by an undoped spacer layer with a thickness of 25 nm. Subsequently to the quantum dots, 300 nm of undoped GaAs were grown that serve as a depletion region in which photogenerated minority charge carriers (holes) will drift towards the DBQW. Here, they can be captured within the adjacent QDs. The sample was finalized with a 50 nm thick GaAs collector contact region with a doping concentration of $n=2\times 10^{19}$ cm^−3^.

In this paper, we demonstrate the ability of Equations (1)–(3) to accurately determine current step heights needed for single photon detection. Figure 3a shows the current density–voltage characteristic of the studied sample up to 4.5 V. Because of the rather large quantum well, multiple resonances can be observed, at 0.5 V, 2.5 V, and 4 V. Under illumination with <100 fW light power only, the photocurrent increases sharply, and large amplitude fluctuations indicate multiple simultaneous charge carrier trapping and escape events that are merged because of the limited time resolution of the setup. The step-like nature can be seen more clearly in the long-term decay of the photocurrent, when the light was switched off. Here, discrete current steps can be observed due to the escape of single charge carriers. Based on the theoretical estimation of Equation (2) and the measured j(V) characteristics, one can calculate the expected step height for such a single charge event, which is shown in Figure 3c. From the heterostructure design and doping profile, as well as from the measured quantization energies (extracted from the photoluminescence spectrum), the leverage factor is estimated to be between $0.03<n_{RTD}<0.06$, which mark the two lines labelled as theory as an upper and a lower bound, respectively. The experimental results extracted from the photocurrent measurements at different bias voltages are shown as red crosses and provide a good agreement with the theory.

## 3. Practical Device Design Considerations

### 3.1. Quantum Dots Are Not a Necessity

Even though there is no strict requirement for quantum dots adjacent to or within the resonant tunneling structure, single-photon detection in RTDs has been exclusively demonstrated in devices containing quantum dots. This might be surprising, considering that there are several advantages in using quantum wells instead of quantum dots for minority charge carrier trapping.

In contrast to QDs that are distributed heterogeneously in position and energy, QWs form a well-defined homogenous layer across the growth surface, which allows for the reproducible fabrication of individual devices. The use of semiconductors such as GaAsSb can provide a deeper electric confinement potential of the quantum well in the valence band, while leaving the conduction band profile nearly unaffected. Stronger confinement of the photogenerated minority charge carriers is assumed to enable operation at even higher temperatures.

**Figure 4:**
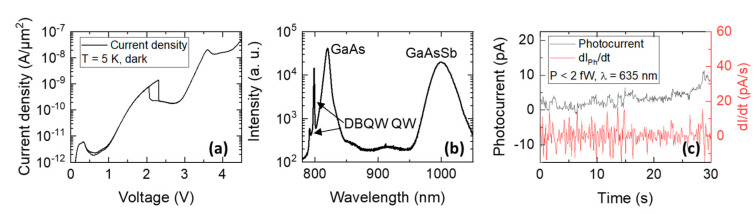
RTD single-photon detector with GaAsSb quantum well as minority charge carrier trap. (**a**) Current density-voltage characteristics measured in the dark at T=5 K. (**b**) Photoluminescence spectrum showing four distinct peaks corresponding to emission from the double-barrier quantum well, bulk GaAs, and the GaAsSb quantum well. (**c**) Photocurrent-time trace taken at V=2.00 V for an incident light power of P<2 fW. Discrete current steps of $\Delta I_{1}\approx 2$ pA indicate single-photon detection events.

To verify our hypothesis that single-photon detection is possible in RTDs without quantum dots, we grew an RTD with a heterostructure that is identical to that of Figure 3, except that is has a 4 nm wide GaAsSb QW instead of the InGaAs QD layer. Similar current density-voltage characteristics with three resonances (see Figure 4a) demonstrate the negligible impact of the GaAsSb layer on the electron transport. The photoluminescence spectrum of Figure 4b shows four distinct emission peaks that can be attributed to emission from the quasibound states of the DBQW (*e-lh* at 790 nm and *e-hh* at 800 nm), the GaAs emitter and collector regions, and the GaAsSb quantum well. Similar to the QD-RTD, the QW-RTD was operated at V=2.00 V and the photocurrent–time trace was measured as a function of time for an incident light power of P<2 fW (see Figure 4c). From the measured j(V) characteristics and Equation (2), the expected current step height is $\Delta I_{1}<3$ pA. Indeed, in the photocurrent–time trace, there seem to be discrete current steps apparent with a step height of $\Delta I_{1}\approx 2$ pA indicating single-photon detection events.

### 3.2. Detector Architectures and Device Design

**Figure 5:**
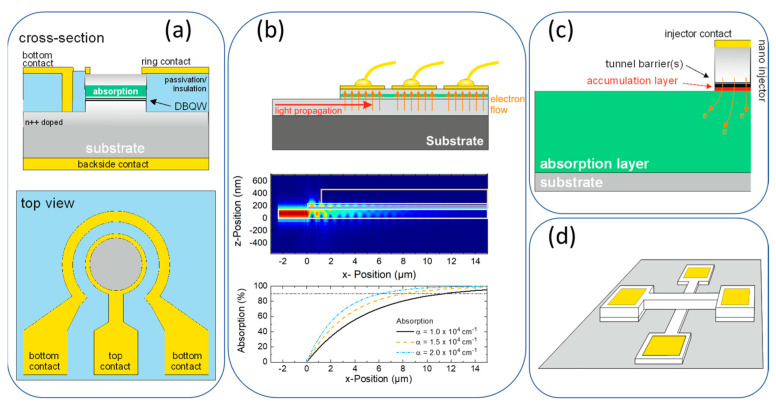
Comparison of different device geometries. (**a**) Ring contact geometry for easy optical access and fabrication. (**b**) Waveguide-integrated RTD photodetector with the photon propagation perpendicular to the electrical current flow. (**c**) Nano-injector with separated optically and electrically active area. (**d**) Cross-wire architecture for sub-µm^2^ size active areas. Figure 5 was originally published in Ref. [22].

We showed that µm to sub-µm device geometries are favorable to ensure the electrical signal generated by a single photogenerated charge carrier is greater than the current noise (see Equation (3)) [21]. At the same time, RTD photodetectors suffer from a low detection efficiency that originates from a low absorption probability and hence quantum efficiency. Devices with large optical volume (large cross-section and thick absorption layers) would be beneficial for the optical properties. The dilemma of conflicting optical and electronic size requirements has been addressed by different device designs. The four most prominent device designs are depicted in Figure 5 and discussed in the subsequent section.

Since predominantly minority charge carriers created in the collector-side depletion region contribute to the photodetection mechanisms, we highly recommend the incorporation of a lower bandgap absorption layer within the collector depletion region independent of the chosen detector architecture. The other epilayers should be chosen to have a bandgap energy larger than the photon energy of the targeted wavelength.

#### 3.2.1. Ring Contact

The ring-contact architecture is amongst the most used device geometries for RTD photodetectors. It is closely related to the standard LED or VCSEL design. An RTD mesa structure is defined by dry- or wet-chemical etching and an annular, ring-shaped contact is applied on top of the RTD mesa structure. The main advantage of the ring-contact architecture is its relative ease of fabrication with which RTDs with a µm^2^-sized active area can be manufactured and contacted. For the smallest devices, the optical aperture can cut-off a large fraction of the incident light power. Due to the relatively high reflection at the semiconductor-air interface and the short interaction length for incident light, the ring-contact geometry suffers from a low quantum efficiency. Higher quantum efficiencies can be achieved by integrating the RTD photodetector into a dielectric photonic crystal structure, such as a distributed Bragg reflector cavity [32,43].

#### 3.2.2. Integrated Waveguide

In the integrated waveguide geometry, the light propagation occurs along an optical waveguide core and perpendicular to the electronic transport. A high quantum efficiency and detector bandwidth can be achieved simultaneously. RTDs integrated in optical waveguides were first employed as high-speed and low-voltage electro-absorption modulator [44,45,46]. Later, RTD integrated waveguide photodetectors have been applied in more sophisticated schemes, such as optical injection locking [47] or self-synchronized optoelectronic oscillators [48], making use of their enhanced functionality due to the NDC region [29].

#### 3.2.3. Nano-Injector

Inspired by the rod cells of the human eye [49], the nano-injector geometry was introduced to solve the challenge of different size requirements for the electrical and optical design of nanoscale phototransistors [50]. The tunneling junction that acts as sensing element is spatially separated from large-area absorption region. High quantum efficiencies and an enhanced gating of the majority charge carrier current can be reached simultaneously. The detection of weak light pulses down to a few photons has been demonstrated, and the nano-injector is considered particularly apt for imaging applications and focal plane arrays [51]. The nano-injector design has only been used for devices in the InP/InGaAs material system, even though there are no apparent restraints from its application in other semiconductor material systems. The majority of nano-injection photodetectors comprises single-barrier diodes [52,53,54] but have also been demonstrated for resonant tunneling structures [55]. The majority of photodetectors in the nano-injector geometry have a built-in electric field, which facilitates that even at very low bias voltages the dominant transport mechanism of photogenerated charge carriers is carrier drift. By the engineering of the collector doping profile, the gain-bandwidth product of these detectors can be tuned [56].

#### 3.2.4. Cross-Wire Structure

The RTD cross-wire geometry was introduced as a method to fabricate diodes with sub-µm^2^-sized active areas [57]. Cross-wire structures are fabricated in a multiple-step selective etching process. The top wire is defined first. The bottom wire perpendicular to the top wire is defined second. The active area $A_{RTD}=l_{b}\cdot l_{t}$ is defined by the junction where top and bottom wire intersect, where $l_{b}$ ($l_{t}$) is the width of the bottom (top) wire. RTDs in cross-wire geometry provide a good optical access since there are no metallic contacts in the vicinity to the active area that could block incident light [58,59,60]. However, the main motivation to fabricate RTD single-photon detectors in cross-wire geometry is their comparably small $A_{RTD}$ (see, e.g., Refs. [10,61,62]). Cross-wire RTD photodetectors also suffer from several disadvantages. The wires contribute significantly to the ohmic resistance, therefore increasing the RC-time constant and limiting the bandwidth [63]. Furthermore, their quantum efficiency is often strongly reduced as only a fraction of the incident light couples to the detector.

### 3.3. Strategies against the Quantum Efficiency Dilemma: Cavity-Enhanced Detectors

One of the most severe limitations of RTD single-photon detectors and RTD photodetectors in general has been their comparably low detection (quantum) efficiency of only a few percent, or even less [10,20,63,64,65]. The main reason for these low detection efficiencies is that only a fraction of the incident light is absorbed and can subsequentially generate a hole that is captured for accumulation in one of the QDs. The low photon absorption sets the upper bound for the RTD single-photon detection efficiency. Typically, the optically active regions that can contribute to a capturing event are between 20 nm and 500 nm thick.

**Figure 6:**
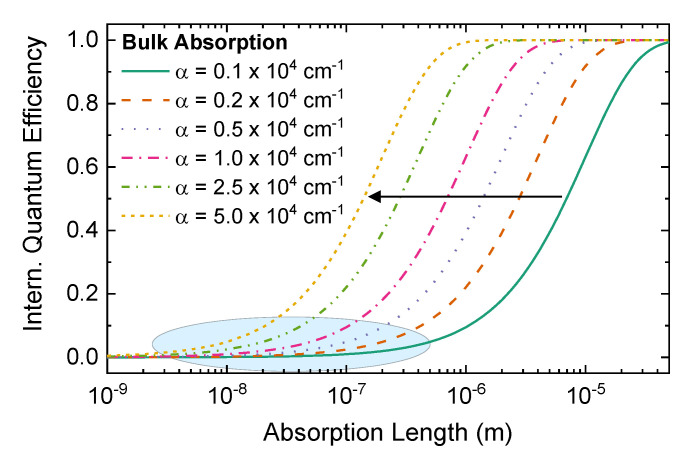
Internal quantum efficiency as a function of absorption length for increasing absorption coefficients from $\alpha =0.1\times 10^{4}$ cm^−1^ to $\alpha =5\times 10^{4}$ cm^−1^. The grey-shaded region represents typical values covered by RTD photodetectors and RTD single-photon detectors. Dependent on the absorption coefficient value, absorber lengths of a few hundreds of nm up tp tens of µm are required to achieve almost unitary internal quantum efficiencies.

Figure 6 shows the maximum internal quantum efficiency given by the bulk absorption calculated for increasing absorption coefficients from $\alpha =0.1\times 10^{4}$ cm^−1^ up to $\alpha =5\times 10^{4}$ cm^−1^ plotted against the absorption length (on the logarithmic scale). The blue-shaded area marks the region covered by RTD photodetectors and single-photon detectors [30,32,34,35,39,66]. To achieve single-photon detection efficiencies η>90%, increasing the absorption layer thickness is neither sufficient nor feasible. The required thicknesses of 1–10 µm introduce too many disadvantages, such as an increased operation voltage, lower speed of operation, and possibly impact ionization processes.

**Figure 7:**
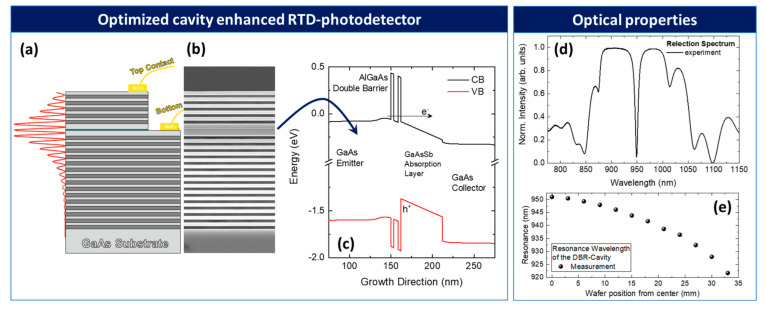
(**a**) Layer structure of the cavity-enhanced RTD photodetector. The RTD-SPD is integrated in an optical resonator formed of five top and fifteen bottom DBR mirror pairs. The GaAsSb absorption layer (shown in blue) is placed at the center of the cavity in the optical field maximum to ensure optimal absorbance. (**b**) Cross-sectional SEM image with dark contrast regions corresponding to the Al-rich containing layer. (**c**) Calculated conduction and valence band profile of the RTD photodetector with GaAsSb absorber. (**d**) Experimentally measured reflectivity. The resonance wavelength is at 950 nm. (**e**) Wafer position-dependent resonance wavelength measured from the center of the 3-inch wafer to the edge. A shift of the resonance wavelength of about Δλ=30 nm can be achieved.

Cavity-enhanced photodetectors can significantly increase the detection efficiency of a photodetector at a target wavelength [32]. Near-unity efficiency can be reached for critical coupling conditions, that is, when the mirror reflectivities of the cavity are precisely matched to the internal absorption [67]. Figure 7a shows the layer structure of the cavity-enhanced RTD photodetector. The RTD-SPD is integrated into an optical resonator that was designed for a resonance wavelength of λ=950 nm. The optical resonator is formed by a top- and a bottom-distributed Bragg reflector (DBR) consisting of 5 and 15 alternating AlAs/GaAs λ/4 mirror pairs, respectively. DBR mirrors are state of the art and widely used for, e.g., vertical cavity surface emitting laser and in cavity quantum electrodynamics. Because of the small lattice constant difference between AlAs and GaAs and the relatively high refractive index contrast, these structures can be grown easily and reproducible. In the present example, the DBR mirrors form a λ-cavity, which ensures that there is enough room for the heavily n-type-doped emitter and collector contact regions ($n=5\times 10^{18}$ cm^−3^) as well as the DBQW RTS and the subsequent 26 nm thick low-bandgap GaAs_0.8_Sb_0.2_ absorption layer. The GaAsSb absorption layer is placed at the field maximum in the center of the cavity to ensure optimal absorption.

Figure 7b shows the cross-sectional scanning electron microscopy (SEM) image of the cavity-enhanced photodetection system with dark contrast regions corresponding to Al-rich layers and Figure 7c shows the calculated conduction and valence band profile of an RTD photodetector with GaAsSb absorber region. Figure 7d shows the experimentally measured reflectivity spectrum of the cavity-enhanced RTD photodetector. The resonance wavelength is at 950 nm. The reflection dip at resonance drops to below 10%, which indicates the almost ideal critical coupling conditions under which quantum efficiencies above 90% can be reached. Figure 7e shows resonance wavelength as a function of the wafer position from the center of the 3-inch wafer to the edge. The semiconductor growth rate of the molecular beam epitaxy chamber decreases from the center to the edge, causing a pronounced blue shift of about Δλ=30 nm.

**Figure 8:**
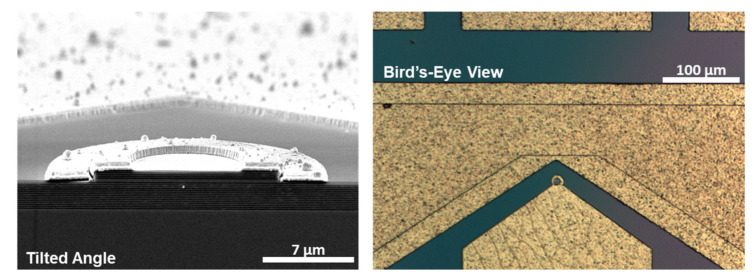
(**Left**) Scanning electron microscopy image of the cavity-enhanced RTD photodetector in ring-contact geometry taken at tilted angle. Electron microscopy image of an RTD photodetector with intracavity ring-shaped contacts. (**Right**) Optical microscopy image of the surface of the sample. The circular shaped contact is clearly visible.

The DBR cavity-enhanced RTD photodetector can readily be fabricated in ring-contact geometry. Figure 8 shows a tilted-angle cross-sectional SEM image. The top DBR mirror is doped, whereas the bottom DBR mirror is left undoped to reduce loss. As a result, the bottom contact is applied as intra-cavity contact. The optical microscopy image in Figure 8 shows the surface of the sample. The circular ring contact is clearly visible. To prevent the ring contact from forming a short circuit with the bottom part of the cavity, the sample surface and mesa-sidewalls were passivated with SiO_2_. The passivation was removed where the bottom intra-cavity contact was applied. The oxide-open areas beneath the bottom contact are clearly visible from the optical microscopy image.

## 4. Quantifying the Device Performance of RTD-SPD

In this section, we assess the performance of RTD single-photon detectors in terms of spectral range, deadtime, dark count rate, detection efficiency, timing jitter, and the ability to resolve photon number. These are the figures of merit that are used in *Hadfield’s* review article on single-photon detectors for optical quantum information applications to compare different detector technologies against each other [68].

### 4.1. Spectral Range

RTD-SPDs have been realized on both GaAs and InP substrates, therefore covering the visible and near-infrared spectral range including the telecommunication wavelengths [10,64]. With RTDs from III-nitrides and the 6.1-Å family, single-photon detection should be achievable in the ultraviolet and mid-infrared spectral regions, respectively.

### 4.2. Deadtime

The deadtime $\tau_{d}$ is the time during which the detector system is unable to reliably register a second photon [68]. The deadtime of a detector determines the maximum detection frequency $1/\tau_{d}$. In SNSPDs, the deadtime is related to the breakdown of the superconductivity subsequent to the absorption of a photon. In SPADs, the deadtime can be artificially prolonged to suppress after-pulsing. RTD-SPDs are theoretically deadtime-free in the classical sense (see Figure 2b) because there is no breakdown of the current that would require a reset of the detector into its initial state. RTD-SPDs can, however, be driven into saturation if the incident photon flux is greater than the escape rate of captured minority charge carriers. The lifetime of these charge carriers depends on various parameters, such as heterostructure, applied bias voltage, or temperature, and can be as high as tens of seconds (in case of quasi-bound states, e.g., in quantum dots) or as low as nanoseconds [18,19]. External electronic reset operations (short reverse bias pulses) can be applied to circumvent the lifetime limitation of the maximum sustainable operation frequency [62].

### 4.3. Dark-Count Rate

The dark-count rate in RTD single-photon detectors has been among the lowest reported for any single-photon detector [68]. Values as low as $2\times 10^{-3}$ s^−1^ have been demonstrated with negligible reduction in detection efficiency [10]. The low achievable dark-count rate brings RTD-SPDs into the spotlight for an application in the telecommunication windows as SPDs and SPADs usually suffer from dark-count rates in the kHz range.

RTD dark counts can be divided into virtual dark counts due to current noise, and actual dark counts due to minority charge carriers that had not been generated in a photon absorption process.

Dark counts due to current noise become relevant in the phototransistor mode, when the current increment induced by a captured minority charge carrier is of the same order of magnitude as the shot noise or near the resolution limit of the read-out electronics.

Actual dark counts are caused by minority charge carriers that originate from a process that does not involve photoexcitation due to the absorption of an incident photon. At low operation voltages, the main source is the intrinsic generation recombination current in the collector-side depletion region. For operation voltages exceeding $q_{0}V≳1.5\;E_{gap}$ (with $E_{gap}$ the bandgap energy), minority charge carrier generation due impact ionization will become dominant [69,70]. Hence, by operating RTD SPDs at cryogenic temperatures and below the threshold for impact ionization, it is possible to eliminate the main sources of actual dark counts and achieve the record low dark count rates [10].

### 4.4. Detection Efficiency

The detection efficiency is the probability of a single incident photon to cause a registered detection event. The RTD quantum efficiency sets an upper bound to the detection efficiency but can further be limited by the read-out circuit. When using differentiating electronics to convert the current step into a trigger pulse, (see Figure 2b), the discriminator level must be chosen appropriately. A low discriminator level ensures that all photon-detection events are recorded at the cost of an increasing dark-count rate.

### 4.5. Timing Jitter

RTDs are amongst the fastest electronic devices that have been manufactured by humankind. As such, they promise fast and precise detection, that is, a low timing jitter. Owing to the large RC-time constant of the circuit and the cross-wire geometry, the first RTD SPDs showed a relatively large timing jitter of about 150 ns [10]. However, recent advances on detectors in nano-injector geometry demonstrated transit-time limited timing jitters down to 15 ps and below [71], which is comparable to state-of-the-art SNSPDs.

### 4.6. Photon-Number Resolution

RTD single-photon detectors are inherently capable of photon-number resolution. The PNR capability of RTDs was already evident in the photocurrent–times traces shown in the pioneering work of Ref. [10], yet there are surprisingly few studies that take on PNR in RTDs in greater detail. Remarkable, RTDs have been demonstrated to keep their PNR even at elevated temperatures of T≤77 K [20].

## 5. Conclusions

RTD single-photon detectors offer a unique and rich platform for photon counting with plenty of uncharted territory open for discovery to the curious researcher.

## Data Availability

The data presented in this study are available upon reasonable request from the corresponding author.
